# SUSANS With Polarized Neutrons

**DOI:** 10.6028/jres.110.030

**Published:** 2005-06-01

**Authors:** Apoorva G. Wagh, Veer Chand Rakhecha, Makus Strobl, Wolfgang Treimer

**Affiliations:** Solid State Physics Division, Bhabha Atomic Research Centre, Mumbai 400085, India; Berlin Neutron Scattering Center, Hahn-Meitner-Institut, Glienicker Strasse 100, 14109 Berlin, Germany

**Keywords:** Bonse-Hart camera, magnetic agglomerates, polarized neutrons, USANS

## Abstract

Super Ultra-Small Angle Neutron Scattering (SUSANS) studies over wave vector transfers of 10^–4^ nm^–1^ to 10^–3^ nm^–1^ afford information on micrometer-size agglomerates in samples. Using a right-angled magnetic air prism, we have achieved a separation of ≈10 arcsec between ≈2 arcsec wide up- and down-spin peaks of 0.54 nm neutrons. The SUSANS instrument has thus been equipped with the polarized neutron option. The samples are placed in a uniform vertical field of 8.8 × 10^4^ A/m (1.1 kOe). Several magnetic alloy ribbon samples broaden the up-spin neutron peak significantly over the ±1.3 × 10^–3^ nm^–1^ range, while leaving the down-spin peak essentially unaltered. Fourier transforms of these SUSANS spectra corrected for the instrument resolution, yield micrometer-range pair distribution functions for up- and down-spin neutrons as well as the nuclear and magnetic scattering length density distributions in the samples.

## 1. Introduction and Discussion

A neutron beam with an extremely sharp angular profile is required in Ultra-Small Angle Neutron Scattering (USANS) studies. Bonse and Hart [1] proposed multiple Bragg reflections from a channel-cut single crystal to obtain a beam with a nearly rectangular angular profile. The Bonse-Hart proposal was first realized in its totality with triple-triple Darwin reflections from optimally designed [2] monochromator and analyzer crystals to achieve the sharpest angular profile [3] for a neutron beam. This has paved the way for Super Ultra-Small Angle Neutron Scattering (SUSANS) studies. SANS experiments with polarized neutrons [4] have measured size distributions of magnetic nanoparticles in samples. Micrometer-sized magnetic agglomerates can be characterized with a polarized SUSANS facility. We present here the first polarized SUSANS instrument.

**Fig. 1:**
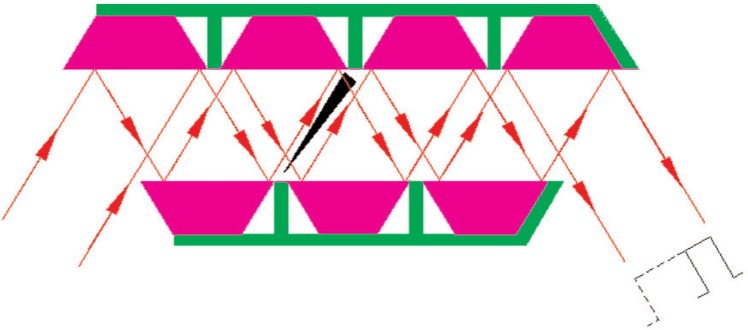
Si prism narrows reflectivity curve by a factor of 3.

**Fig. 2:**
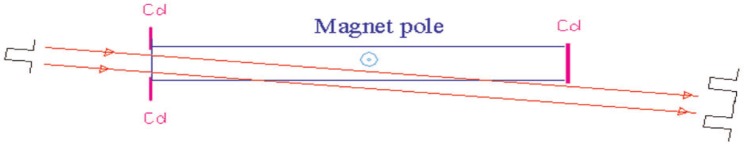
Magnetic prism separates up- and down-spin states.

**Fig. 3:**
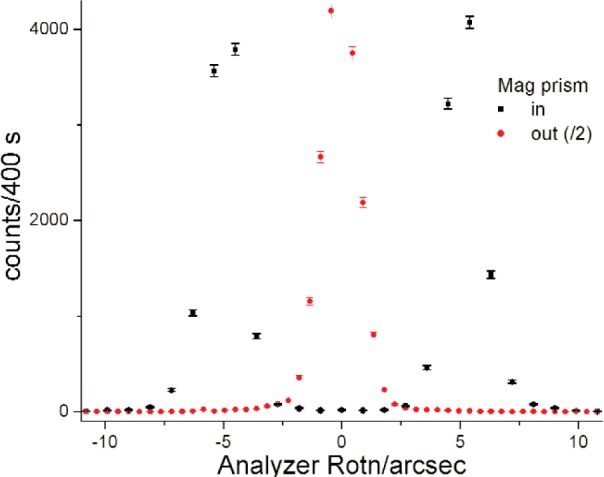
Up-down spin splitting with a magnetic prism.

The instrument has been set up at the V12b Double Crystal Diffractometer of the 10 MW reactor at the Hahn-Meitner-Institut in Berlin, Germany. Neutrons of 0.54 nm wavelength were subjected to 7 Ewald reflections each at the monochromator and analyzer channel-cut silicon single crystals. Side faces of the reflector slabs were cut parallel to the exiting beams and covered with absorber Cd foils (Fig. 1) as prescribed by Wagh [2] to eliminate spurious tails in the reflectivity curve. In each crystal, a silicon prism was inserted after the third reflection (Fig. 1) to deflect neutrons by about 4 arcsec. Due to this shift between the triple and subsequent fourfold reflection patterns within each channel-cut crystal, the width of the rocking curve for the unpolarized beam (central peak in Fig. 3), obtained by rotating the analyzer crystal, reduced to nearly 2 arcsec [5]. Between the monochromator and analyzer, the horizontal neutron beam traversed a vertical magnetic field of 29 × 10^4^ A/m (3.7 kOe) in a 2 cm high air gap between 2 cm × 20 cm rectangular poles of a C-shaped permanent magnet, at a small angle to the diagonal of the rectangle (Fig. 2). Neutrons were thus deflected by the “magnetic air prism” [6] of 90° apex angle. The magnet was fabricated by attaching 8 rare earth permanent magnet slabs (2 cm wide, 5 cm long and 1.25 cm high, *BH*_max_ > 30 MGOe) each, just above the upper pole piece and just below the lower pole piece, within a magnet-grade soft iron “C”. The neutron angle of incidence to the magnet was optimized to achieve a separation of about 10 arcsec between the up-and down-spin neutron peaks [7] and a neutron count rate of about 10/s at each peak position (Fig. 3) for a 10 mm wide and 20 mm high beam. A sample could be inserted in a holder, in a 8.8 × 10^4^ A/m (1.1 kOe) vertical magnetic field produced by a pair of ferrite magnets, placed between the magnetic prism and analyzer. The SUSANS instrument was thus equipped with the polarized neutron option.

The neutron polarization attained here is ideal, since unlike other polarizers, a magnetic prism separates the two polarizations with 100 % efficiency and the spin-flip probability for either state during its passage to the sample through *air* in the guide field is insignificantly small. Hence the polarization *P* and flipping efficiency *ε* can both [4] be safely equated to unity. We further have an advantage of recording SUSANS spectra for both the spin states side by side in a single rocking curve. This enables a direct comparison between the up- and down-spin spectra with no need for separate normalizations or for a spin-flip operation.

**Fig. 4:**
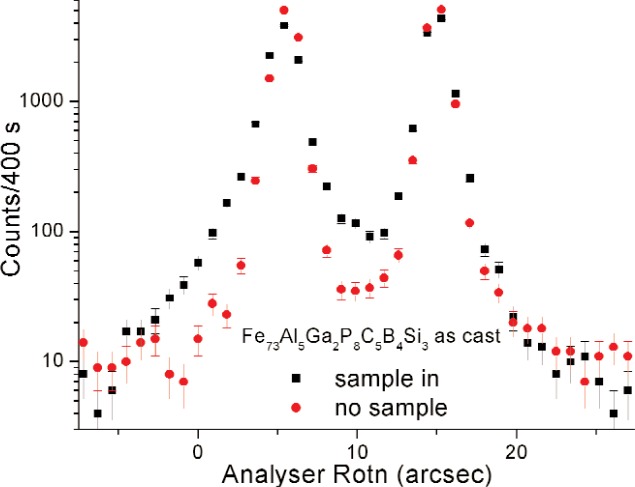
Polarized SUSANS for a magnetic sample.

We illustrate the capability of the instrument with polarized SUSANS spectra of an as-cast Fe_73_Al_5_Ga_2_P_8_C_5_B_4_Si_3_ ribbon sample (Fig. 4). The rocking curve recorded without a sample, representing the instrument resolution, is also shown for comparison. The sample has broadened the up-spin peak (left) considerably, but has an insignificant effect on the down-spin peak.

This measurement needs to be combined with complementary techniques in order to characterize the distributions over shapes, sizes and orientations of nuclear and magnetic scattering structures in the sample. Proper Fourier transforms of scattering length distributions taking all these variations into account should then be used to analyze the polarized SUSANS spectra. However a flavor of the information obtainable can be provided even by simplistically assuming identical spherically symmetric nuclear and magnetic structures. The up- and down-spin SUSANS spectra corrected for the instrument resolution yield squares of the respective Fourier transforms, whose spherically symmetric inverse Fourier transforms help visualize the averaged spatial distributions, as outlined below.

**Fig. 5:**
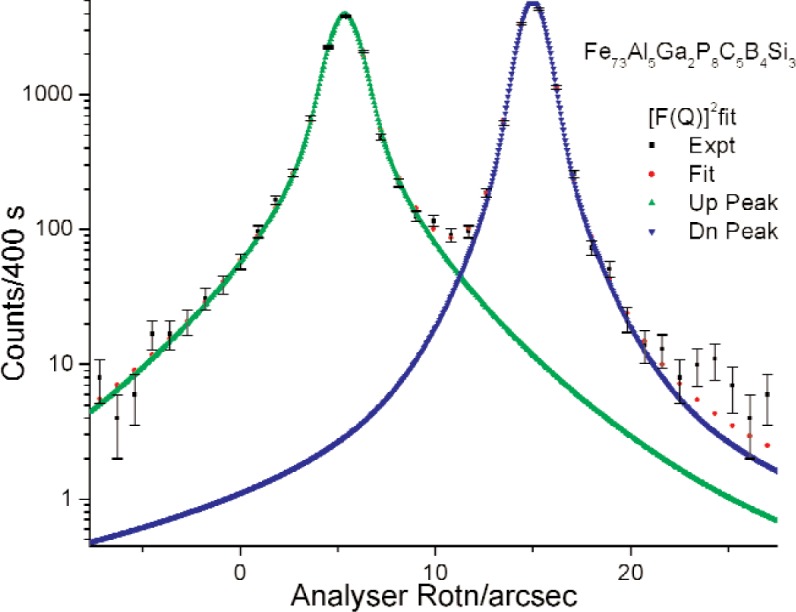
Fitted up- and down-spin SUSANS spectra.

**Fig. 6:**
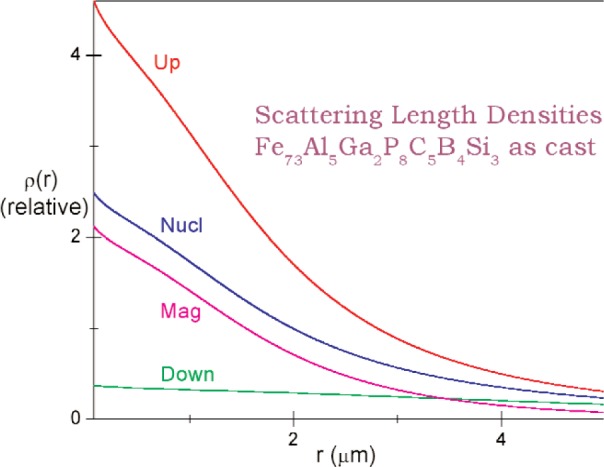
Scattering length densities.

**Fig. 7:**
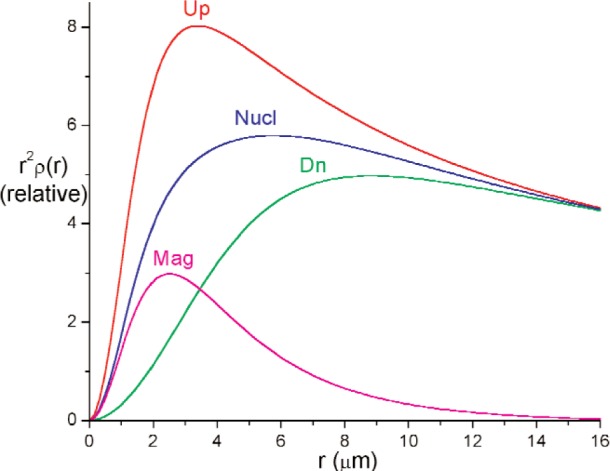
Scattering length shell densities.

**Fig. 8:**
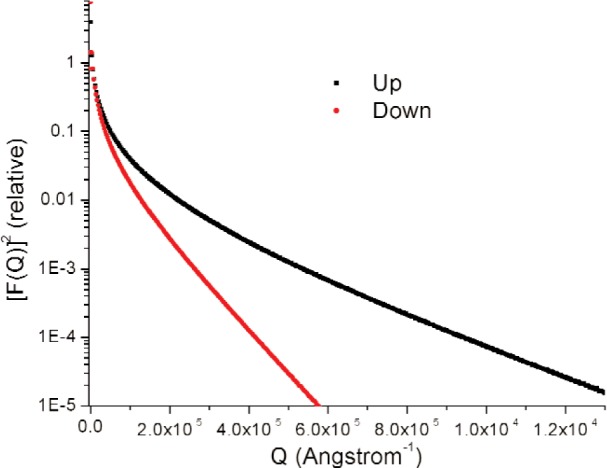
|*F*u(*Q*)|2, |*F*_d_(*Q*)|2corrected for instrument resolution.

**Fig. 9:**
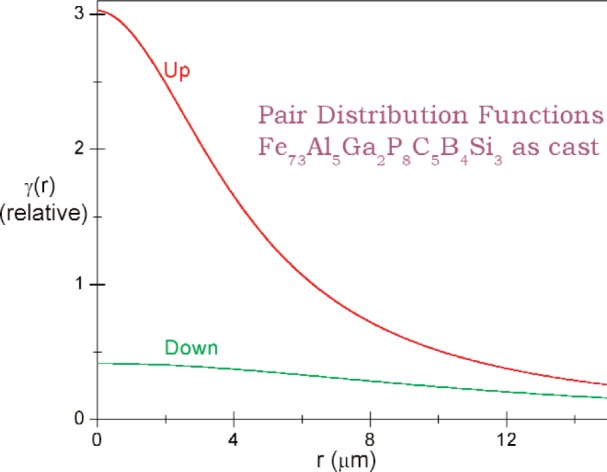
Pair distribution functions.

We assume parameterized spherically symmetric scattering length distributions *ρ*_u_(r) and *ρ*_d_(*r*) of identical, spherically symmetric “particles” for up- and down-spin neutrons respectively. Each Fourier transform *F*(*Q*) equals the volume integral [8] of the respective *ρ* (*r*)exp(–*i****Q*** · ***r***), ***Q*** denoting the wave vector transfer. The sum of convolutions of |*F*_u_(*Q*)|^2^ and |*F*_d_(*Q*)|^2^ with the respective fits to the instrument resolution, peaked at parameterized *Q*-centers, is least-square fitted (Fig. 5) to the sample SUSANS spectrum to extract *ρ*_u_(*r*) and *ρ*_d_(*r*). The nuclear and magnetic scattering length density distributions *ρ*_N_(*r*) and *ρ*_M_(*r*) then equal [4,8] half the sum and difference respectively between *ρ*_u_(*r*) and *ρ*_d_(*r*) (Fig. 6). The distributions extend upto a few micrometers, the up-spin distribution being narrower but stronger at *r* = 0 than that for the down-spin, as expected from the SUSANS spectra. The average diameter deduced for the scattering “particle” is about 3 µm with the up- and 9 µm with the down-spin neutrons. Fig. 7 displays the scattering length densities over a shell of radius *r*. The magnetic shell density is narrower and weaker than the nuclear shell density. The fitted |*F*(*Q*)|^2^ distributions (Fig. 8) are inverse Fourier transformed [8] to obtain the respective pair distribution functions *γ* (*r*) (Fig. 9), which are wider than the respective *ρ* (*r*) curves.

To recapitulate, the first polarized SUSANS instrument spanning *Q* > 10^–4^ nm^–1^ range and capable of characterizing micrometer-sized magnetic agglomerates in samples, has been commissioned.
